# Triple blockade of Ido-1, PD-L1 and MEK as a potential therapeutic strategy in NSCLC

**DOI:** 10.1186/s12967-022-03730-y

**Published:** 2022-11-22

**Authors:** Carminia Maria Della Corte, Vincenza Ciaramella, Kavya Ramkumar, Giovanni Vicidomini, Alfonso Fiorelli, Valerio Nardone, Salvatore Cappabianca, Immacolata Cozzolino, Federica Zito Marino, Gaetano Di Guida, Qi Wang, Robert Cardnell, Carl Michael Gay, Davide Ciardiello, Erika Martinelli, Teresa Troiani, Giulia Martini, Stefania Napolitano, Jing Wang, Lauren Averett Byers, Fortunato Ciardiello, Floriana Morgillo

**Affiliations:** 1 Medical Oncology, Department of Precision Medicine, University of Campania ‘Luigi Vanvitelli’, Naples, Italy; 2 Department of Thoracic Head and Neck Medical Oncology, The University of Texas MD Anderson Cancer Center, Houston, TX USA; 3 Thoracic Surgery Unit, Department of Traslational Sciences, University of Campania ‘Luigi Vanvitelli’, Naples, Italy; 4 Radiology and Radiotherapy, Department of Precision Medicine, University of Campania ‘Luigi Vanvitelli’, Naples, Italy; 5 Pathology Unit, Department of Mental and Physical Health and Preventive Medicine, University of Campania ‘Luigi Vanvitelli’, Naples, Italy; 6 Department of Department of Bioinformatics & Computational Biology, The University of Texas MD Anderson Cancer Center, Houston, TX USA

**Keywords:** Ido-1, Checkpoint inhibitor, Resistance, EMT

## Abstract

**Background:**

Despite the recent progress in the treatment and outcome of Non Small Cell Lung Cancer (NSCLC), immunotherapy has still significant limitations reporting a significant proportion of patients not benefiting from therapy, even in patients with high PD-L1 expression. We have previously demonstrated that the combined inhibition of MEK and PD-L1 in NSCLC patients derived three dimensional cultures exerted significant synergistic effect in terms of immune-dependent cancer cell death. However, subsequent experiments analyzing the expression of Indoleamine 2,3-dioxygenase-1 (Ido-1) gene expression demonstrated that Ido-1 resulted unaffected by the MEK inhibition and even increased after the combined inhibition of MEK and PD-L1 thus representing a potential escape mechanism to this combination.

**Methods:**

We analyzed transcriptomic profile of NSCLC lung adenocarcinoma cohort of TCGA (The Cancer Genome Atlas), stratifying tumors based on EMT (Epithelial mesenchymal Transition) score; in parallel, we investigated the activation of Ido-1 pathway and modulation of immune cytokines productions both in NSCLC cells lines, in peripheral blood mononuclear cells (PBMCs) and in *ex-vivo* NSCLC spheroids induced by triple inhibition with an anti-PD-L1 monoclonal antibody, the MEK inhibitor and the Ido-1 inhibitor.

**Results:**

In NSCLC lung adenocarcinoma patient cohort (from TCGA) Ido-1 gene expression was significantly higher in samples classified as mesenchymal according EMT score. Similarly, on a selected panel of NSCLC cell lines higher expression of MEK and Ido-1 related genes was detected in cells with mesenchymal phenotype according EMT score, thus suggesting a potential correlation of co-activation of these two pathways in the context of EMT, with cancer cells sustaining an immune-suppressive microenvironment. While exerting an antitumor activity, the dual blockade of MEK and PD-L1 enhances the secretion of pro-inflammatory cytokines (IFNγ, TNFα, IL-12 and IL-6) and, consequently, the expression of new immune checkpoints such as Ido-1. The triple inhibition with an anti-PD-L1 monoclonal antibody, the MEK inhibitor and the Ido-1 inhibitor demonstrated significant antiproliferative and proapoptotic activity on *ex-vivo* NSCLC samples; at the same time the triple combination kept increased the levels of pro-inflammatory cytokines produced by both PBMCs and tumor spheroids in order to sustain the immune response and simultaneously decreased the expression of other checkpoint (such as CTLA-4, Ido-1 and TIM-3) thus promoting an immune-reactive and inflamed micro-environment.

**Conclusions:**

We show that Ido-1 activation is a possible escape mechanism to immune-mediated cell death induced by combination of PD-L1 and MEK inhibitors: also, we show that triple combination of anti-PD-L1, anti-MEK and anti-Ido-1 drugs may overcome this negative feedback and restore anti-tumor immune response in NSCLC patients’ derived three dimensional cultures.

**Supplementary Information:**

The online version contains supplementary material available at 10.1186/s12967-022-03730-y.

## Background

By the better understanding of the interaction between the immune system and tumor cells, treatment of non-small cell lung cancer (NSCLC) has recently been implemented with the introduction of immune checkpoint inhibitors, such as monoclonal antibodies directed against cytotoxic T-lymphocyte-associated antigen-4 (CTLA-4) and programmed cell death protein-1 (PD-1)/programmed cell death ligand-1 (PD-L1) pathway, which boost the host’s own immune response against cancer [1]. In particular, anti-PD-1/PD-L1 antibodies have demonstrated to be effective strategies by inducing significant durable tumor responses in some subgroups of patients. The only predictive biomarker for immunotherapy response used for patient’s selections in clinical setting is PD-L1 expression [1, 2]. Actually, immunotherapy has still significant limitations as monotherapy reporting a significant proportion of patients not benefiting from therapy, even in patients with high PD-L1 expression.

Clinical data have shown the potentiality of genomic markers to negatively predict immunotherapy response as with oncogene-addicted NSCLCs (EGFR, ALK, ROS1 mutant) and KRAS/STK11 mutant tumors [1, 2]. Moreover, translational studies highly suggest that epithelial-to-mesenchymal-transition (EMT) is associated with different immune profiling, including high expression of targetable immune-checkpoints (PD-L1, CTLA-4, Ido-1, TIM3, LAG3) [3]. However, the relation between EMT and regulation of immune microenvironment is very complex and not fully elucidated: cancer cells with mesenchymal features sustain immune-suppressive microenvironment [4], favoring expansion of CD4 + T-cells and reducing CD8 + T-cells effectors infiltration [5].

There is a strong rationale for combining targeted therapy and immune checkpoint blockade in the treatment of cancers. Inhibition of the MAPK pathway, which is a key intracellular pathway implicated in tumorigenesis, by MEK inhibitors (MEK-I), may have pleiotropic effects on the tumor immune microenvironment. In particular, preliminary studies show how MEK inhibition results in increased tumor antigen presentation, promotes T-cell infiltration of the tumor and regulates a number of cytokines, enhancing tumor recognition by the immune system. We have previously demonstrated that MEK signal regulates transcription of mRNA levels of PD-L1 and the combined inhibition of MEK and PD-L1 in NSCLC patients derived three dimensional cultures exerted significant synergistic effect in terms of immune-dependent cancer cell death [6]. This synergism is the result of both direct cancer cell toxicity by MEK-I and the immune-stimulatory effect of MEK-I on cytokine secretion profile of cancer cells and peripheral blood mononuclear cells (PBMCs) which sustain an immune-reactive and inflammatory microenvironment. Mechanistically, this last aspect is amplified even more by the re-activation of T cells by anti-PD-L1 drugs. In addition, the blockade of MEK and PD-L1 also modified the immune checkpoints gene expression on spheroids as we detected a decrease of PD-L1, CTLA-4, TIM3 and LAG3 thus indicating a potential role of MEK also on T cells exhaustion [6].

**Fig. 1:**
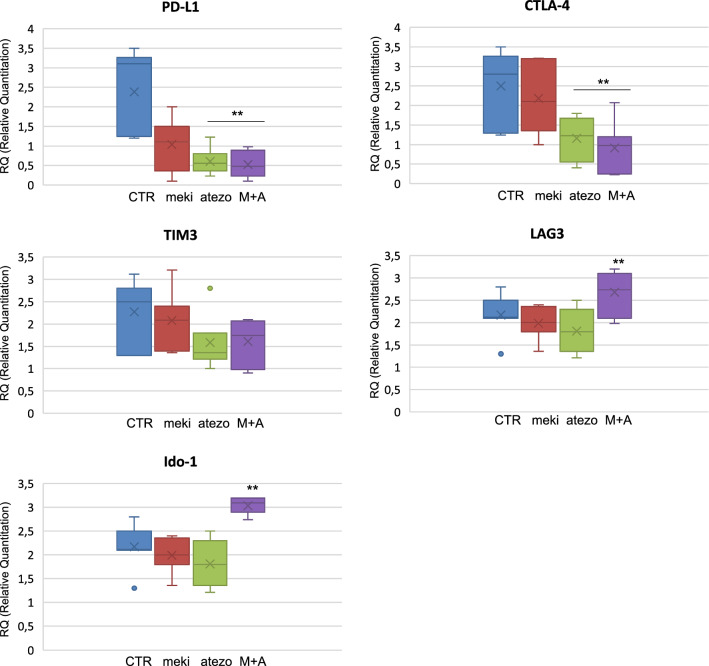
Real time qPCR analysis of immune checkpoint genes as PD-L1, CTLA4, TIM3, and Ido-1 expressed by spheroids untreated (Control) or treated with selumetinib (Mek-i), atezolizumab (anti-PD-L1) or their combinations. Results were normalized to 18S mRNA and analyzed by ΔCt method. One-way ANOVA test followed by Tukey’s test were used for statistical analysis. **p < 0.01

However, subsequent experiments in this setting led us to also analyze the expression of Indoleamine 2,3-dioxygenase-1 (Ido-1) gene expression in the list of immune checkpoints, which instead resulted increased after the combined inhibition of MEK and PD-L1 (Fig. 1). Specifically, our previous study [6], showed the changes in cytokines gene expression in NSCLC patients’ derived three-dimensional cultures after treatment with MEK and PD-L1 inhibitors (Fig. 1) with an increase of IFNγ, IL-12, IL-6 and TNFα: although the increase of this group of cytokines is able to create a favorable microenvironment for inflammatory and immune response, on the other side it can enhances feedback signals such as the upregulation of other immune checkpoints that are not dependent from the MEK pathway, such as Ido-1 (Fig. 1). However, at the same time targeted therapy and immune checkpoint inhibitors affect cytokine production from both cancer cells and PBMCs. Moreover, other previous studies uncovered data on specific interplay between MEK signaling and Ido-1 activation in dendritic cells in tumor microenvironment [7] and on activation of Ido-1 expression by MEK-inhibitors in melanoma patients [8].

Ido-1 is a natural immune-regulatory enzyme initially thought to be solely implicated in the modulation of innate immune responses during infection, subsequent discoveries demonstrated Ido-1 as a mechanism of acquired immune tolerance [9]. It is involved in the first, rate-limiting step to kynurenine of the tryptophan, which is an essential amino acid with both neuropsychological as well as immunological functions. Ido-1 expressing dendritic cells suppress antigen-specific T-cell responses directly or indirectly through activating regulatory T cells. In the settings of cancer, many different types of cells express Ido-1, including tumor and stromal cells, in variable proportions. In this context, the expression of Ido-1 protects premalignant cells from immune surveillance and tumor cells from anticancer drugs [9]. Ido-1 may represent one of the new potential target for these tumors, since it is not only expressed by cancer cells, but also by tumor associated microenvironment. Specifically, macrophages expressing Ido-1 behave as immunosuppressive cells producing TGFβ, and IL-10 which decrease CD8 + cytotoxic T-cells infiltration and express themselves PD-L1, thus supporting the rationale to combine anti-PD-L1 therapy with Ido-1 inhibitors [9]. Also, in NSCLC Ido-1 is highly related to other immune-suppressive immune checkpoint expression, like PD-L1 [5]. Thus, targeting Ido-1 is an attractive novel immunotherapy approach [10].

In this work, we investigate the activity of the combination of the MEK-inhibitor, with checkpoint inhibitors targeting PD-L1 and Ido-1 in NSCLC patients’ derived models. In particular, considering that tumor cells are most likely exposed to cytokines produced by tumor infiltrating lymphocytes, we studied the effects of the triple blockade of MEK, PD-L1 and Ido-1 on patients’ derived three dimensional samples, using tumor spheroids from NSCLC patient's biopsy, generated as previously described [6]. Preserving cancer and immune cells, this system allows us to study in vitro both the anti-tumor and immune effects of anti-cancer drugs on all components of the tumor.

## Methods

### Human NSCLC cell line and drugs peripheral blood mononuclear cells (PBMCs) and spheroids isolation

Human NSCLC cell lines were obtained by the American Type Culture Collection (ATCC, Manassas, Virginia, USA) and were cultured in RPMI-1640 (Sigma-Aldrich) medium which was supplemented with 10% foetal bovine serum (FBS; Life Technologies, Gaithersburg, Maryland, USA) in a humidified atmosphere with 5% CO_2_. The identity of cell lines was confirmed by human STR testing (ATCC). Selumetinib (MEK-I, AZD6244) and atezolizumab were purchased from Selleck Chemicals, Munich, Germany. Epacadostat was provided by Incyte Biosciences International S.a.r.l.

Primary antibodies for western blot analysis against phospho-MEK, MEK, phospho-MAPK44/42, MAPK44/42, PD-L1, Ido-1, CTLA4, LAG-3, TIM-3 and MHC-I were obtained from Cell Signaling Technology; the following secondary antibodies from Bio-Rad were used: goat anti-rabbit IgG, rabbit anti-mouse IgG and monoclonal anti-α tubulin antibody from Sigma Chemical Co.

### Peripheral blood mononuclear cells (PBMCs) and spheroids isolation

Human samples and biopsies were collected after obtaining a written informed consensus from patients in accordance with the Declaration of Helsinki. The use of these samples for research purposes was approved by our local Ethical Committee and all patients gave their written informed consent to the use of the tumor sample. All below described methods were performed in accordance with guidelines and regulations. PBMCs from NSCLC patients were isolated by Ficoll-Paque Plus (GE Healthcare). Isolated cells were grown for 24 h or 5 days in complete medium composed by RPMI 1640 containing human AB serum (10%), Ultraglutamine I (1%), penicillin and streptomycin (1%) along with beads coated with anti-CD3 and anti-CD28 (Life Technologies) at a ratio of 1 bead per 10 cells. We developed a protocol to generate *ex-vivo* 3D cultures from lung cancer patient samples. All fresh tumor tissue samples were kept on ice and processed in sterile conditions on the day of collection. Tissue fragments were digested as previously described [6] in a 37 °C shaker at low to moderate speed (e.g. 200 rpm) for incubation time for 1-2 h and cells were separated with serial centrifugation. For 3D cultures, cells were seeded in complete medium with 5% matrigel in order to preserve three-dimensional structure.

### Western blot analysis

Protein lysates were obtained by homogenization in RIPA lyses buffer [0.1% sodium dodecylsulfate (SDS), 0,5% deoxycholate, 1% Nonidet, 100 mmol/L NaCl, 10 mmol/L Tris–HCl (pH7.4), 0.5 mmol/L dithiotritol, and 0.5% phenylmethyl sulfonyl fluoride, protease inhibitor cocktail (Hoffmann-La Roche)] and clarification by centrifugation at 14,000 rpm for 15 min a 4 °C. Protein samples containing comparable amounts of proteins, estimated by a modified Bradford assay (Bio-Rad), were subjected to western blot and immune-complexes were detected with the enhanced chemiluminescence kit ECL plus, by Thermo Fisher Scientific (Rockford, IL) using the ChemiDoc (Bio-Rad). Each experiment was done in triplicate.

### Quantitative real time PCR

Total RNA was extracted using Trizol reagent (Life Technologies). Reverse transcriptase reaction was carried out to convert 1 μg of isolated RNA into cDNA using Sensi fast reverse transcriptase (bioline) according to the manufacturer instruction. Expression levels of genes encoding for: IFNγ, IL-12, IL-1β, IL-8, IL-10, TNFα, TIM-3, CTLA-4 were analyzed using Real time quantitative PCR (RT-qPCR). Amplifications were done using the SYBR Green PCR Master Mix (Applied Biosystems). The thermal cycling conditions were composed of 50 °C for 2 min (step 1) followed by a denaturation step at 95 °C for 10 min (step 2) and then 40 cycles at 95 °C for 15 s and 60 °C for 1 min (step 3). All samples were run in duplicate, in 25 μL reactions using a quant studio 7 flex (Applied Biosystems) and relative expression of genes was determined by normalizing to 18S, used as internal control gene; to calculate relative gene expression in value it was used the 2- ΔCt or 2- ΔΔCt method. Nonspecific signals caused by primer dimers were excluded by dissociation curve analysis and use of non-template controls.

### Flow cytometry

For FACS surface staining, cells were washed in staining buffer (SB) (2% FBS; 0,1% sodium azide in PBS) and after a blocking of 10 min with SB + Ab serum 20%, were stained for 30 min with mouse monoclonal antibodies. The antibodies used were: anti- PD-1/PD-L1, Ido-1, TIM3, LAG3 and CTLA-4 (Miltenyi Biotec). Stained cells were washed 2 times, resuspended in SB and then acquired on a FACS ACCURI C6 (BD Biosciences).

### Assessment of apoptosis

Apoptosis was evaluated by flow cytometry using AnnexinV-FITC and 7-Amino-Actinomicin D (7-AAD) double staining (Thermo fisher) according to the manufacturer’s instruction. The detection of viable cells, early and late apoptosis cells, and necrotic cells were performed by BD Accuri™ C6 (BD Biosciences) flow cytometer and subsequently analyzed by ACCURI C6 software (Becton Dickinson). Results represent the median of three separate experiments, each performed in duplicate.

### Immunofluorescence

Organoids in matrigel were fixed for 20 min with a 4% paraformaldehyde (PFA) solution and made permeable for 10 min with 0.1% Triton X-100 in phosphate-buffered saline (PBS) at room temperature. Then organoids were incubated with a specific mouse monoclonal Ab raised against Ido-1 (1:1000 in blocking solution, 3% BSA in TBS-Tween 0.1%, Sigma) for 2 h at 37 °C followed by revelation using Alexa Fluor 488-conjugated anti-rabbit IgG antibodies (Jackson Immunoresearch Laboratories, West Grove, PA, USA) at a dilution of 1:1000 for 1 h. The fluorescence was analyzed by an LSM-410 Zeiss confocal microscope.

### Viability assay

Organoids were seeded in 96-well plates at the density of 1 × 2000 cells/well and were treated with Atezolizumab and Anti-Ido-1 in single agent and their combinations, for 6 days. Cell proliferation and cytotoxicity was measured with MTS Assay Kit (Abcam) according to manufacturer’s instructions. The MTS assay protocol is based on the reduction of the MTS tetrazolium compound by viable cells to generate a colored formazan dye that is soluble in cell culture media. The formazan dye is quantified by measuring the absorbance at 490–500 nm.

### TCGA analysis

Comparison of gene expression of selected genes was performed as previously described [11]. EMT score was calculated according to previously published method [12].

### Statistical analysis

Statistical analysis was performed using Graphpad Prism Software V.6.0 (Graphpad Software, San Diego, California, USA). Quantitative data were reported as mean ± Standard Deviation (SD) from three or more independent experiments. Data were compared with one-way Analysis Of VAriance (ANOVA) statistical test followed by Tukey’s test or unpaired t-test; p values less than 0.05 were considered statistically significant.

## Results

### Role of MEK signal on immune checkpoint expression

**Fig. 2:**
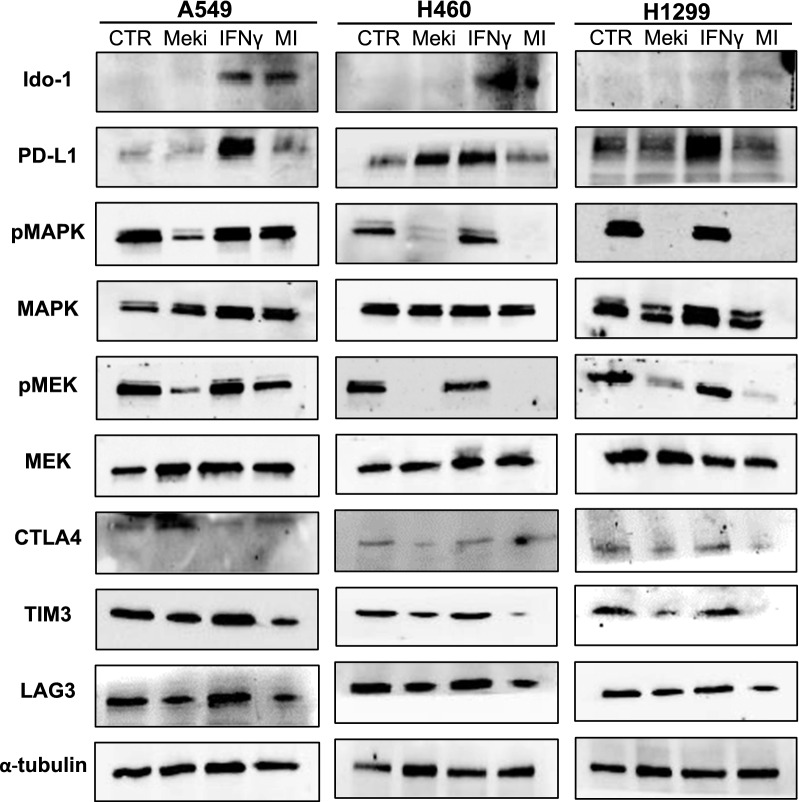
Western blot analysis of Ido-1, PD-L1, MAPK, phospho-MAPK (pMAPK), MEK, phospho-MEK (pMEK), CTLA4, TIM3 and LAG3 on protein lysates from NSCLC cell lines A549, H460 and H1299. α-tubulin was included as a loading control. Control: untreated control, Mek-I: selumetinib, IFNγ: interferon-γ

We have previously demonstrated that PD-L1 is transcriptionally activated by MEK pathway in cancer cells and that inhibition of MEK and PD-L1 is effective as potential anticancer strategy [6]. We therefore asked if MEK pathway may influence activation of other immune checkpoint molecules and in particular of Ido-1; we analyzed the protein expression levels of MAPK, MEK, PD-L1 and Ido-1 in a panel of NSCLC established cell lines harboring WT or mutant *KRAS* gene (A549 and H460, *KRAS* mutated (G12C/G12S/Q61H) and H1299, *KRAS* WT) after IFNγ stimulation and after treatment with the MEK-inhibitor, selumetinib (Fig. 2).

IFNs are known to be potent immune check point-inducers. As tumor-infiltrating lymphocytes (TILs) are the predominant source of IFNγ, they might upregulate negative feedback signals, hereby potentially contributing to tumor immune escape.

IFNγ stimulation induced an increased protein expression of both pMEK/pMAPK activation and of PD-L1, and to a lesser extent also of CTLA-4, TIM, LAG and Ido-1. Based on the known regulatory role of MEK signal on PD-L1 transcription [6], the effect on PD-L1 is counteracted by the treatment with selumetinib. Similarly, also CTLA-4, TIM3 and LAG3 protein expression is reduced by MEK-inhibition even in presence of IFN γ; on the contrary, IFNγ stimulated-Ido-1 expression was not modified and thereby results independent from MEK activation status. This observation supports the potential role of Ido-1 as a potential escape mechanism to the dual blockade of PD-L1 and MEK, as shown in Fig. 1, and opens a chance for a combined triple treatment strategy targeting MEK pathway, PD-L1 and Ido-1.

### Activation of Ido-1 is correlated to EMT in NSCLC

Since we have previously demonstrated that Ido-1 expression is highly correlated with other immune-suppressive markers in NSCLC (such as PD-1 and PD-L1) [3] and that NSCLC tumors undergone to epithelial-mesenchymal-transition (EMT) are enriched for those markers [12], we hypothesized that NSCLC with EMT features could be the best models to explore Ido-1 signaling.

**Fig. 3:**
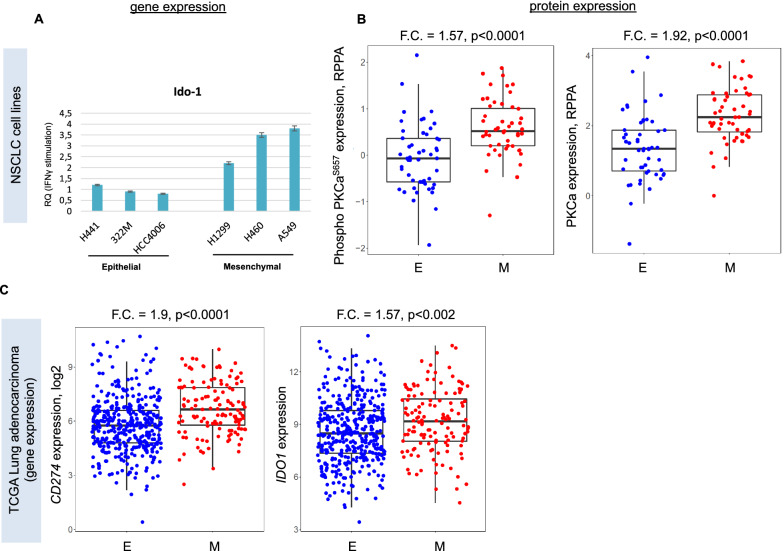
**A** mRNA expression level of *IDO-1* in a panel of NSCLC cell lines: epithelial (E) cells (H441, 322 M, HCC4006) versus mesenchymal (M) cells (H1299, H460, A549). Results were normalized to 18S mRNA and analyzed by ΔCt method. **B** Comparison of phosphorylated PKCa and total levels between E and M NSCLC cell lines. Fold change in expression and p-value by t-test are indicated. **C** Comparison of *IDO1* and *CD274* mRNA expression between E and M tumors in the TCGA LUAD cohort. Fold change in expression and p-value by t-test are indicated

First, we investigated in vitro the changes in *IDO-1* gene expression (measuring mRNA levels by PCR) induced by IFNγ stimulation, in a panel of lung cancer cells, classified as epithelial or mesenchymal, according previously published EMT score [12]. A comparison between the 3 epithelial (E) NSCLC cell lines (H441, 322 M, HCC4006) and the 3 mesenchymal NSCLC cell lines (H1299, H460, H549) showed a significantly higher level of IFN-γ stimulated *IDO-1* mRNA induction in the second group (Fig. 3A). Next, we compared the proteomic profile of E versus M cell lines as measured by reverse phase protein array (RPPA). Interestingly, among the differentially expressed proteins between the two groups, higher levels of Protein kinase C alpha (PKCa), a known positive upstream regulator of Ido-1 expression [13], was detected in the M cell lines (p < 0.0001; Fold Change = 1.92). Similarly, protein levels of the phosphorylated form of PKCa was also significantly higher in the M cell lines (p < 0.0001; Fold Change = 1.57) (Fig. 3B). Similarly, NF-ΚB (both total and phosphorylated form), another known activator of Ido-1 [13], was higher in M cells (p < 0.05). These results suggest that intracellular pathways involved in Ido-1 expression are active in cell lines that underwent EMT. Since Ido-1 expression in cancer is modulated by interaction with stromal and immune cells, we extended our analysis to tumor samples interrogating the clinical dataset of TCGA NSCLC cohort [3].

First, we compared gene and protein expression levels in the sub-cohort of lung adenocarcinoma (LUAD) tumors from TCGA [3] between E and M tumors. We found higher gene expression of *IDO-1* and *CD274* (PD-L1) (p < 0.01; Fold change > 1.5) in M LUADs, thus suggesting a potential association between these two immune pathways and the EMT process (Fig. 3C). Also, gene expression of *IDO-1* was positively correlated with PD-L1 protein expression (Pearson’s rho > 0.3, p < 0.0001) in TCGA LUAD tumors (Additional file 1: Figure S1A). Similar results were also confirmed in the NSCLC cell lines (Additional file 1: Figure S1B).

Thus, these results suggest a concomitant activation of PD-L1 and Ido-1 pathways in NSCLC tumors that have undergone EMT, that may be explored as novel targets.

### Effects of triple blockade of MEK, PD-L1 and Ido-1 on patients’ derived PBMCs

**Fig. 4:**
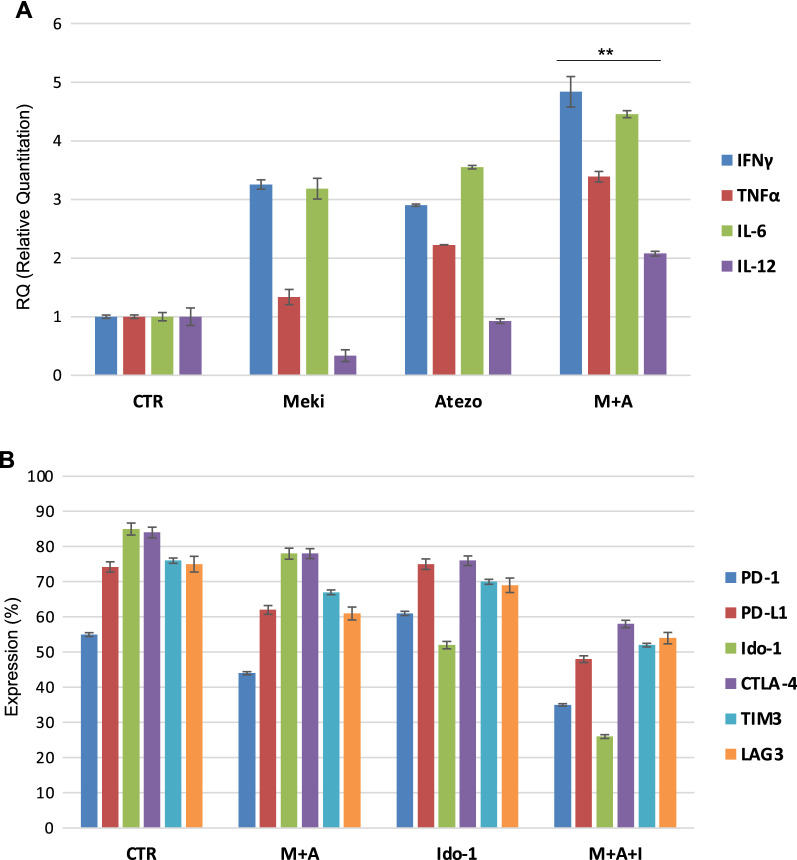
**A** Real time qPCR analysis of IFNγ, TNFα, IL-6 and IL-12 expressed by mRNA from PBMC before (Control) and after treatment with selumetinib (Mek-I), Atezolizumab (ant-PD-L1) alone and in combination. Results were normalized to 18S mRNA and analyzed by ΔCt method. One-way ANOVA test followed by Tukey’s test were used for statistical analysis (** *p* < 0.01). **B** Flow cytometry analysis of immune checkpoint molecules as PD-1/PD-L1, Ido-1, CTLA-4, TIM3 and LAG3 in PBMCs surface before (Control) and after treatment with selumetinib plus atezolizumab, Ido-1 inhibitor (anti-Ido-1) alone and in triple combination

To further explore the effect of combined targeted therapy and immune checkpoint inhibitors on cytokine production, we also evaluated the expression levels of several cytokines produced by NSCLC patients’ derived PBMCs before and after treatment in vitro with the PD-L1 inhibitor, atezolizumab, and the MEK inhibitor, selumetinib. The dual inhibition sustained an increased production of IFNγ, TNFα, IL-12 and IL-6 by PBMCs (Fig. 4A), that contribute to expression of Ido-1 on cancer cells.

Then, we investigated if the addition of the Ido-1 inhibitor to the dual blockade of MEK and PD-L1 may affect the expression of immune checkpoints and therefore can block potential escape mechanisms. PD-1/PD-L1, CTLA-4, TIM3, LAG3 and Ido-1 expression were tested on PBMCs surface through FACS analysis (after permeabilization), before and after treatment with MEK-I, selumetinib, PD-L1 inhibitor, atezolizumab, and the Ido-1 inhibitor, epacadostat, alone and in combinations. The triple combination of drugs reduced the levels of PD-L1, TIM3, LAG3, Ido-1 and CTLA-4 (Fig. 4B) as compared to control.

### Effects of triple blockade of MEK, PD-L1 and Ido-1 on patients’ derived three dimensional cultures

We generated tumor spheroids from NSCLC patient's biopsy, as previously described [6].

**Fig. 5:**
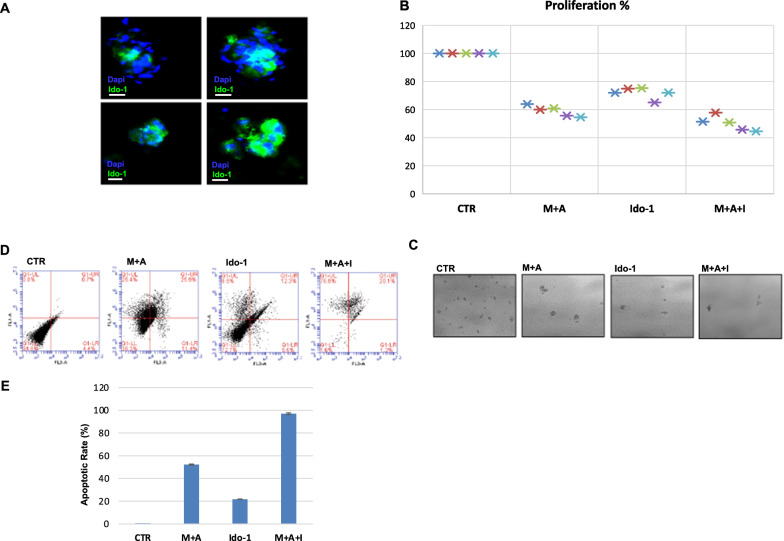
**A** Localization by immunofluorescence of Ido-1 in patient-derived tumour spheroids. Images represent different tumour spheroids deriving from the same patient sample: 4′,6-diamidino-2-phenylindolefluorescent nuclear staining (blue), Ido-1 staining (green). Scale bar: 10 µm. **B** MTS cell proliferation assay in human spheroids (n = 5) after 6 days of treatment with MEK-inhibitor, selumetinib, anti-PD-L1 drug, atezolizumab, and Ido-1 inhibitor as single agents and in combination. One-way ANOVA test followed by Tukey’s test were used for statistical analysis **p < 0.01. **C** Representative images of one sample. **D** Effect of drug treatment on induction of apoptosis in three dimensional spheroid cultures. Representative flow cytometric analysis of apoptosis. One representative experiment is shown. **E** Histogram of data expressed as median percentage of apoptotic cells in 5 samples

First, we confirmed by immunofluorescence staining the expression of Ido-1 in these models. We detected Ido-1 expression as a widely diffuse staining in both tumor and stromal immune cells (Fig. 5A).

To evaluate the anti-tumor effect of the triple blockade of MEK, PD-L1 and Ido-1, we treated 5 established ex vivo cultures with atezolizumab, selumetinib, or epacadostat or their combination for 6 days. Treatment with single drug epacadostat exerted a limited anti-proliferative effect accounting for a percentage of surviving cells > 70% across all samples (Fig. 5B–C). Despite the heterogeneity of the response between patients, the triple combination of MEK, PD-L1 and Ido-1 inhibitors exerted the strongest effect with a further 15% increase in tumour cell death over the double combination of MEK and PD-L1 inhibitors.

We further demonstrated, by annexin V detection, that the strong effect of the triple combination of MEK, PD-L1 and Ido-1 inhibitors on cell death is mostly accompanied by apoptosis (Fig. 5D, E). Results show a significant increase of apoptotic cells among the 5 ex vivo cultures analysed: the percentage of apoptosis reaches the 50% in the double combination of MEK-I with anti-PD-L1 or Ido-1-I, and even increased up to a peak of 90% in the triple drug combination.

**Fig. 6:**
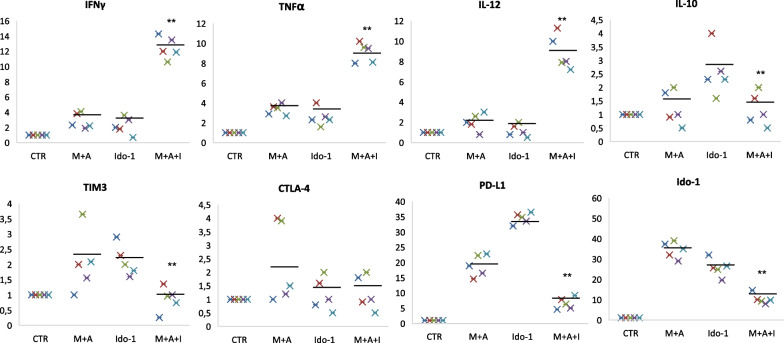
Real time qPCR analysis of IFNγ, TNFα, IL-12, IL-10, TIM3, CTLA-4, PD-L1 and Ido-1 expressed by mRNA from three dimensional spheroid cultures before (Control) and after treatment with MEK-inhibitor, selumetinib, anti-PD-L1 drug, atezolizumab, and Ido-1 inhibitor as single agents and in triple combination. Results were normalized to 18S mRNA and analyzed by ΔCt method. One-way ANOVA test followed by Tukey’s test were used for statistical analysis (**p < 0.01)

To better characterize the effect on PD-L1 and Ido-1 immune signaling, we evaluated changes in gene expression of PD-L1 and *IDO-1* after 72 h’ treatment with the triple combination, by RT-PCR (Fig. 6). Treatment with selumetinib, atezolizumab and epacadostat determined a significant reduction in expression of PD-L1 and *IDO-1* compared to single agents, suggesting the clear involvement of these molecules in the regulation of antitumor systems.

Finally, we also explored the modifications in cytokines and other immune checkpoints gene expression by RT-PCR on spheroids after treatments and we detected an increase of IFNγ, IL-12 and TNFα especially by triple combination of MEK, PD-L1 and Ido-1 inhibitors and a simultaneous decrease of CTLA-4 and TIM-3 (Fig. 6) thus indicating a potential effect on T cells exhaustion.

## Discussion

In our previous work we demonstrated the anti-tumor activity of dual targeting of an immune-checkpoint, such as PD-L1, and an intracellular target, such as MEK. [6] In the present manuscript, we show that Ido-1 activation is a possible escape mechanism to immune-mediated cell death induced by combination of PD-L1 and MEK inhibitors: also, we show that triple combination of anti-PD-L1, anti-MEK and anti-Ido-1 drugs may overcome this negative feedback and restore anti-tumor immune response in NSCLC patients’ derived three dimensional cultures.

Other groups showed that treatment with MEK inhibitor in melanoma patients increases Ido1 expression [8], and this is coherent with the known interplay between MAPK pathways and Ido-1 in dendritic cells [7]. We hypothesized that the activation of Ido-1 represents an escape mechanism to the combination of PD-L1 and MEK inhibitors, counterbalancing the immune-mediated cell death. In cancer patients, activation of immune system involves a multitude of redundant signal networks dynamically balanced between immune-mediated cell death, auto-immune reactions and tumor-induced immune-suppression; thus, novel immunotherapy combinations can be designed as novel strategy to sustain anti-tumor immune response.

Ido-1 expression in cancer has been described in a wide variety of cells both at the level of the tumor microenvironment and the peripheral blood. Depending on the exact location of expression, different induction pathways and effector functions have been observed. Several inflammatory cytokines such as IFNγ, IL-1, IL-6, IL-32, TNFα, and TGFβ were evidenced to drive Ido-1 induction. In the different compartments, Ido-1 expression is closely interconnected with other immune checkpoint molecules already targeted by current immunotherapies. In the local tumor microenvironment, CTLA-4 expression in Tregs upregulates Ido-1 in DCs, which reciprocally promotes Treg activation. This interplay of immune checkpoints is also evidenced in the peripheral blood, where Ido-1 expression by PBMCs was demonstrated to be associated with increased circulating PD- L1^+^ CD8^+^ T-cells and CTLA-4^+^ Tregs. Of interest, blockade of CTLA-4 and/or PD-1 has been reported to upregulate Ido-1 expression as a result of the increased IFNγ-production by reactivated effector T-cells. Of interest, in our model, Ido-1, differently from CTLA-4, TIM-3 and LAG-3, is the only checkpoint not influenced by the MEK pathway and therefore the only one able to drive an escape. In preclinical models, Ido-1 blockade has been demonstrated to be effective as part of combination therapy including immune checkpoint therapy, DNA-damaging chemotherapy and radiotherapy [14]. Interestingly, mesenchymal tumors tend to express higher levels of immune-suppressive markers, that are potentially targetable, including Ido-1. Ido-1 activation is implicated in EMT-induced immune-suppression [15], thus representing an interesting novel target, specifically for therapeutic resistant tumors, that often show mesenchymal phenothype according EMT score [12]. Here, we show that tumors with high EMT score express higher levels of Ido-1; similarly, we found that NSCLC cell lines with high EMT score produce higher Ido-1 transcript levels and display activation of PKC protein, a mediator of Ido-1 intracellular activation. These results suggest that modulation of Ido-1 may be of higher relevance in tumors undergone EMT.

In the present study, using *ex-vivo* organoids cultures, we demonstrated a significant synergistic effect in terms of immune-dependent cancer cell death by the combination of MEK-I, anti-PD-L1 and anti-Ido-1 drugs. The addition of epacadostat indeed potentiated tumor cell death, by multiple mechanisms: inducing a robust apoptosis, sustaining an inflammatory environment, lowering T-cell exhaustion.

In the clinical scenario, Ido-1 has been proposed as a target and several Ido-1 inhibitors have been tested in clinical trials. Epacadostat was the first competitive selective Ido-1 inhibitor, with a safe and promising profile in solid tumors in phase 1–2 [16] alone or combination with anti-CTLA-4 ipilimumab [17] and anti-PD-1 pembrolizumab [18]. Unfortunately, the phase III trial in melanoma patients (ECHO- 301/KEYNOTE-252) failed in confirming efficacy of epacadostat and pembrolizumab combination [18], leaving open question on the clinical relevance of Ido-1 inhibition in cancer treatment. In this trial, no biomarkers were used for patient’s selection and neither pharmacodynamics studies were conducted [17]. However, other clinical data from different cohorts of melanoma patients confirm a biological rational for targeting Ido-1. In a cohort of melanoma patients receiving adjuvant treatment with IFN- α2b, levels Kyn/Trp levels, that is a biomarker of Ido-1 activation, were monitored under treatment and they resulted positively increased in plasma from treated patients as compared to untreated cohort [19]. Similarly, melanoma patients who were resistant to previous treatment with radiotherapy plus ipilimumab showed increased plasma Kyn/Trp ratio, further confirming that Ido-1 may be activated as escape mechanism to immunotherapy induced anti-tumor response [19].

Currently, NSCLC patients receive as standard of care anti-PD-1 drug as single agent or in combination with chemotherapy, eventually plus anti-CTLA-4; in this context, potential new strategies of treatment after failure of anti-PD-1/anti-CTLA-4 therapy are needed. Among the combinations that are being investigated for NSCLC patients in early phase clinical trials, combination of MEK inhibitor, binimetinib, and anti-PD-1 agent, pembrolizumab, is under investigation in a Phase I/Ib study in advanced NSCLC (NCT03991819) and triple combination of MEK inhibitor, selumetinib, and tremelimumab (anti-CTLA-4) and durvalumab (anti-PD-L1) has been proposed by a single center, investigator-initiated Phase I/II clinical trial [20]. In the design of both trials, the schedule proposed for combinations include intermittent or continuous dosing of MEK inhibitors, in order to maximize immune mediated anti-tumor efficacy and safety profiling. This strategy highlights the need of futher investigation of the clinical dosing of these drugs, based on the hypothesis immune effects of MEK inhibition may depend on dosing schedule. According to our opinion, one of the main issue of translating preclinical findings on combination of targeted and immunotherapy drugs into clinical setting, is the difficulty of predict the dose-dependent effect of anti-cancer drugs (such as MEK inhibitor) on immune system of patients.

## Conclusions

The results of the present study on patients’ derived *ex-vivo* NSCLC cultures, showing positive anti-tumor immune response induced by the triple combination of Ido-1, PD-L1 and MEK- inhibition, encourage further research in this setting, especially in model with mesenchymal and immune-resistant features. Moreover, regarding clinical scenario, these results may be of high relevance, suggesting novel biomarkers to test in early clinical trials, such as Ido-1 activation in MEK inhibitor plus immunotherapy resistant- NSCLC patients enrolled in clinical trials.

In this context, for preclinical research we believe that *ex-vivo* models represents an individualized model to assess T cell-based therapies being a suitable and useful system to study the immune-dynamics of cancer. Future studies should include ex vivo models derived by immune-resistant NSCLC patients and investigate also other potential targets related to Ido-1 signal, such as the two other Trp-degrading enzymes, IDO-2 and TDO [21].

In conclusion, we foresee that next research on the role of these enzymes on cancer immune escape would be useful to discover new personalized immuno-oncology therapeutic strategies.

## Supplementary Information

**Additional file 1: Figure S1. **Correlation of mRNA expression level of *IDO1 *with protein expression of PD-L1 in TCGA LUAD tumors (A) and NSCLC cell lines (B).

## Data Availability

All data generated or analyzed during this study are included in this published article [and its supplementary information files].
