# Supplementary figures and images for: Triple blockade of Ido-1, PD-L1 and MEK as a potential therapeutic strategy in NSCLC
Source: J Transl Med. 2022 Nov 22;20:541. doi: 10.1186/s12967-022-03730-y (PMC9682755; doi:10.1186/s12967-022-03730-y)

## Suppl. Fig. 1

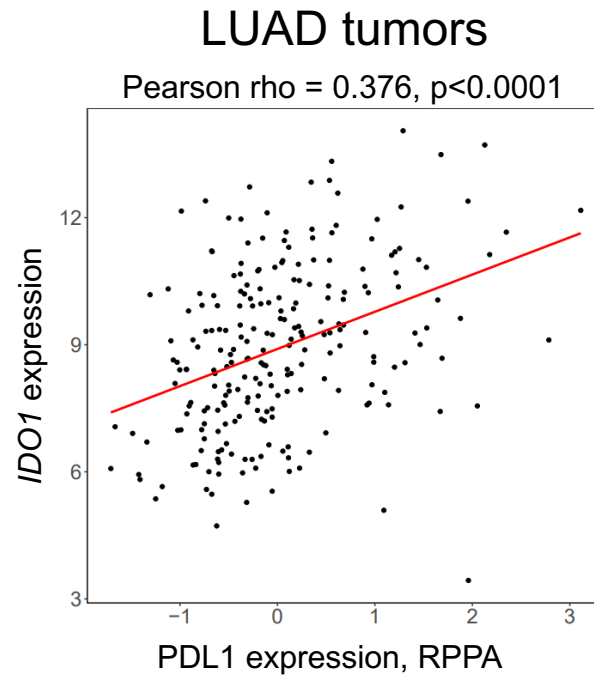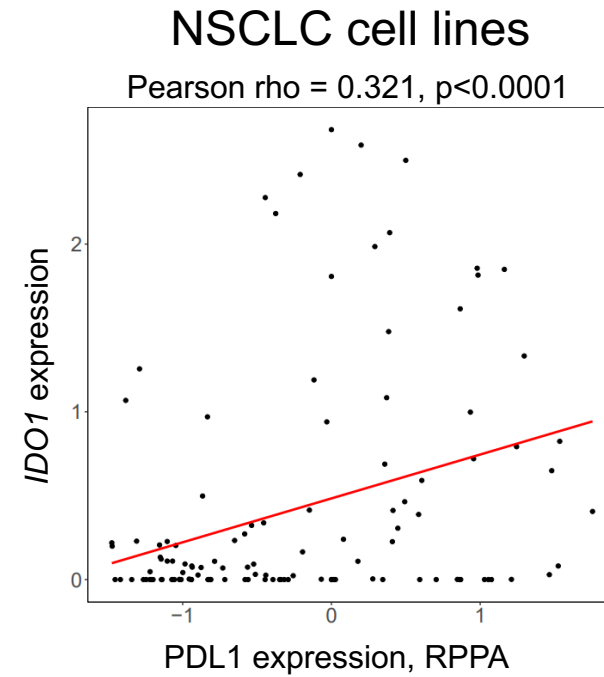

Supplement: Supplementary file 1 — Additional file 1: Figure S1. Correlation of mRNA expression level of IDO1 with protein expression of PD-L1 in TCGA LUAD tumors (A) and NSCLC cell lines (B). [file 12967_2022_3730_MOESM1_ESM.pdf]
